# Increased methylation of lung cancer-associated genes in sputum DNA of former smokers with chronic mucous hypersecretion

**DOI:** 10.1186/1465-9921-15-2

**Published:** 2014-01-09

**Authors:** Shannon Bruse, Hans Petersen, Joel Weissfeld, Maria Picchi, Randall Willink, Kieu Do, Jill Siegfried, Steven A Belinsky, Yohannes Tesfaigzi

**Affiliations:** 1 Lovelace Respiratory Research Institute, Albuquerque, NM, Mexico; 2 Department of Pharmacology & Chemical Biology, Hillman Cancer Center of the University of Pittsburgh Medical Center, Pittsburgh, PA, USA

**Keywords:** Methylation of gene promoters, Persistent cough and phlegm, Sputum DNA, Former smoker, Lung cancer genes

## Abstract

**Background:**

Chronic mucous hypersecretion (CMH) contributes to COPD exacerbations and increased risk for lung cancer. Because methylation of gene promoters in sputum has been shown to be associated with lung cancer risk, we tested whether such methylation was more common in persons with CMH.

**Methods:**

Eleven genes commonly silenced by promoter methylation in lung cancer and associated with cancer risk were selected. Methylation specific PCR (MSP) was used to profile the sputum of 900 individuals in the Lovelace Smokers Cohort (LSC). Replication was performed in 490 individuals from the Pittsburgh Lung Screening Study (PLuSS).

**Results:**

CMH was significantly associated with an overall increased number of methylated genes, with *SULF2* methylation demonstrating the most consistent association. The association between *SULF2* methylation and CMH was significantly increased in males but not in females both in the LSC and PLuSS (OR = 2.72, 95% CI = 1.51-4.91, p = 0.001 and OR = 2.97, 95% CI = 1.48-5.95, p = 0.002, respectively). Further, the association between methylation and CMH was more pronounced among 139 male former smokers with persistent CMH compared to current smokers (SULF2; OR = 3.65, 95% CI = 1.59-8.37, p = 0.002).

**Conclusions:**

These findings demonstrate that especially male former smokers with persistent CMH have markedly increased promoter methylation of lung cancer risk genes and potentially could be at increased risk for lung cancer.

## Background

Chronic obstructive pulmonary disorder (COPD) is predicted to become the third leading cause of death worldwide by 2020 [1]. Prevalence is increasing both in developing and developed countries as a result of tobacco consumption [2,3], environmental exposures such as pollution and biomass fuel smoke [4,5] and the growing elderly population [6]. Clinically, COPD is defined by the presence of poorly reversible airflow obstruction, although this definition simplifies the complex causes and manifestations of the disease [7]. Chronic mucous hypersecretion (CMH), characterized by persistent mucous cell metaplasia in the epithelial layer and submucosal glands of the respiratory tract, is a clinically important COPD phenotype [8]. CMH leads to worse respiratory symptoms, greater susceptibility to respiratory infections, more frequent COPD exacerbations, and increased risk of mortality [9-14].

Numerous publications, and two recent meta-analyses, have determined that prior CMH significantly increases the risk for later lung cancer [15,16]. While smoking clearly contributes to both diseases, analyses controlling for smoking have demonstrated that the association between lung cancer and prior CMH is at least partially independent of smoking [15,16]. It is therefore plausible that CMH and lung cancer have some shared molecular pathology. Previous case–control studies of incident lung cancer assessing the same genes as in the current study demonstrated that promoter methylation of these genes is associated with lung cancer risk [17,18].

The goal of this study was to determine whether there was any association between CMH and prevalence of methylation of promoters in lung cancer-predictive genes in sputum DNA of smokers. Therefore, methylation specific PCR (MSP) was used to assess promoter methylation of eleven genes in sputum samples of smokers from the Lovelace Smokers Cohort (LSC). Replication was performed in smokers from the Pittsburgh Lung Screening Study (PLuSS).

## Methods

### Study populations

This study is approved by the Western Institutional Review Board (Olympia, WA; #20031684) and all subjects signed informed consent for their participation. The catchment area for the LSC was the Albuquerque, NM metropolitan area, comprising a population of approximately 850,000 persons. Inclusion criteria for entry into the current study were age 40 to 75 years, current or former cigarette smoking (with a minimum of 10 pack-years) upon entry into the study, and ability to understand English. The LSC disproportionately enrolled women ever-smokers to study the susceptibility to the development of smoking-related lung diseases since women are underrepresented in most such studies in the United States. Detailed characteristics of the LSC have been described elsewhere [19,20]. From the LSC cohort, 311 non-Hispanic white (NHW) individuals meeting the criteria for CMH were included along with 589 NHW current or former smoking controls. Current and former smoking was assessed by self-report at baseline concurrent with sputum sampling. Former smokers are those who have stopped smoking for at least 2 years prior to self-report.

Study participants for the replication cohort were from the Pittsburgh Lung Screening Study (PLuSS), a volunteer cohort established to investigate lung cancer biomarkers in an at-risk population of smokers which has previously been described [21,22]. From the total cohort (n = 3638), 490 NHW individuals (183 men and 307 women) had information allowing classification with respect to chronic mucous hypersecretion and had provided sputum for DNA isolation. Spirometric testing procedures have previously been described for both the PLuSS and the LSC [19,21].

Because a unifying definition for CMH was not available in both cohorts two criteria were used to define CMH: In the LSC, CMH was defined as present in participants that had self-reported cough productive of phlegm for at least 3 months per year for at least 2 consecutive years (ie. the standard definition of chronic bronchitis). In the PLuSS, CMH was defined as self-reported cough productive of phlegm as assessed by both a first and second questionnaire (with a median questionnaire interval of 3.5 years), and self-reported cough producing phlegm for “most days a week” or “several days a week” in the past year, as assessed by the second questionnaire.

### Methylation specific PCR

Nested MSP assays were used to detect methylation of cytosines at cytosine-phosphate-guanine sites in DNA recovered from the sputum samples, as previously described [23,24]. We studied the promoter methylation of a panel of eleven tumor suppressor genes with previously identified roles in predicting lung cancer risk [17,18]. These genes included *PCDH*-*20* (Protocadherin); *SULF*-*2* (6-O-endosulfatase 2); *GATA* binding protein-4 and −5 transcription factors; *PAX*-*5α* and *PAX*-*5*β (paired box protein transcription factors); *p16*; *MGMT* (O (6)-methylguanine-DNA methyltransferase); *DAPK* (Death-associated protein kinase); *DAL*-*1* (Differentially expressed in adenocarcinoma of the lung); and *JPH*-*3* (Junctophilin). Methylation by this technique was scored positive or negative, as previously described [18].

### Statistical analysis

Chi-square and Fisher’s exact tests were used for the univariate analyses of categorical variables, while two-sample t-tests and Kruskal-Wallis tests were used for continuous variables. For multivariable analyses of CMH, logistic regression was performed. Predictors included gene specific methylation prevalence, and also total methylation (continuous variable representing the sum of genes methylated within an individual). Additional predictors included age, education (dichotomized as at least high school or less than high school education), COPD status, sex, pack-years smoking, and current smoking status. When the LSC and PLuSS were combined for analyses adjustment for cohort was included. Model fitting iterations were performed with the R package glmulti using the small sample size corrected Akiake information criterion to determine best-fitting models [25]. All statistical analyses were performed in R version 2.12.0 or SAS version 9.2.

## Results

### CMH is associated with higher prevalence of gene promoter methylation in smokers

The initial study was conducted in 900 NHW current and former smokers from the LSC with available sputum methylation data. At time of sputum collection, there were 311 smokers with and 589 smokers without CMH. In unadjusted analysis, prevalence of *SULF2* methylation was significantly higher in those with CMH than without CMH (39 % and 30 % respectively, p < 0.01, Table 1). A replication study was performed in the PLuSS, comprised of 140 smokers with and 350 smokers without CMH, and in unadjusted analysis, prevalence of *SULF2* methylation was significantly higher in those with CMH than those without CMH (40 % and 26 % respectively, p < 0.01, Table 2).

**Table 1:** Select variables by CMH status in the LSC

**LSC**	**Total**		**CMH**		**No CMH**		
	**n = 900**	**(100.0)**	**n = 311**	**(100.0)**	**n = 589**	**(100.0)**	
**Characteristic**	**n or mean**	**(%) or (SD)**	**n or mean**	**(%) or (SD)**	**n or mean**	**(%) or (SD)**	**p value**
**Female**	673	(74.8)	222	(71.3)	451	(76.6)	0.088
**Baseline age**	55.9	(9.6)	55.1	(9.8)	56.3	(9.4)	0.067
Education > = HS*	663	(73.7)	207	(66.6)	456	(77.4)	**<0.001**
**Obese**	278	(30.9)	93	(29.9)	185	(31.4)	0.642
**Pack years**	41.1	(20.9)	44.2	(21.8)	39.5	(20.2)	**0.001**
**Baseline smoker**	494	(55.6)	236	(76.4)	258	(44.6)	**<.0001**
**Baseline COPD**	281	(31.2)	123	(39.6)	158	(26.8)	**<.0001**
**Total methylation**	2.47	(2.12)	2.66	(2.22)	2.37	(2.06)	0.057
**PCDH20**	333	(37.0)	125	(40.2)	208	(35.3)	0.149
**SULF2**	299	(33.2)	122	(39.2)	177	(30.1)	**0.005**
**GATA4**	348	(38.7)	125	(40.2)	223	(37.9)	0.495
**PAX5A**	138	(15.3)	46	(14.8)	92	(15.6)	0.743
**p16**	154	(17.1)	61	(19.6)	93	(15.8)	0.147
**MGMT**	249	(27.7)	87	(28.0)	162	(27.5)	0.881
**DAPK**	153	(17.0)	54	(17.4)	99	(16.8)	0.833
**GATA5**	152	(16.9)	55	(17.7)	97	(16.5)	0.643
**PAX5B**	90	(10.0)	30	(9.7)	60	(10.2)	0.797
**DAL1**	71	(7.9)	27	(8.7)	44	(7.4)	0.521
**JPH3**	229	(25.4)	91	(29.3)	138	(23.4)	0.056

*High school.

**Table 2:** Select variables by CMH status in the PLuSS

**PLuSS**	**Total**		**CMH**		**No CMH**		
	**n = 490**	**(100.0)**	**n = 140**	**(100.0)**	**n = 350**	**(100.0)**	
**Characteristic**	**n or mean**	**(%) or (SD)**	**n or mean**	**(%) or (SD)**	**n or mean**	**(%) or (SD)**	**p value**
**Female**	307	(62.7)	90	(64.3)	217	(62.0)	0.637
**Baseline age**	60.3	(6.4)	59.5	(6.1)	60.6	(6.5)	0.075
Education > = HS*	474	(96.7)	134	(95.7)	340	(97.1)	0.422
**Obese**	158	(32.2)	45	(32.1)	113	(32.3)	0.976
**Pack Years**	55.9	(20.1)	59	(19.1)	54.6	(20.4)	**0.029**
**Baseline smoker**	335	(68.4)	113	(80.7)	222	(63.4)	**<0.001**
**Baseline COPD**	238	(48.6)	81	(57.9)	157	(44.9)	**0.009**
**Total methylation**	2	(1.84)	2.26	(2.04)	1.9	(1.75)	0.052
**PCDH20**	135	(27.6)	47	(33.6)	88	(25.1)	0.059
**SULF2**	147	(30.0)	56	(40.0)	91	(26.0)	**0.002**
**GATA4**	166	(33.9)	47	(33.6)	119	(34.0)	0.928
**PAX5A**	68	(13.9)	23	(16.4)	45	(12.9)	0.302
**p16**	92	(18.8)	32	(22.9)	60	(17.1)	0.143
**MGMT**	123	(25.1)	34	(24.3)	89	(25.4)	0.792
**DAPK**	75	(15.3)	21	(15.0)	54	(15.4)	0.905
**GATA5**	64	(13.1)	18	(12.9)	46	(13.1)	0.932
**PAX5B**	33	(6.7)	9	(6.4)	24	(6.9)	0.864
**DAL1**	37	(7.6)	14	(10.0)	23	(6.6)	0.194
**JPH3**	41	(8.4)	15	(10.7)	26	(7.4)	0.235

*High school.

In adjusted analysis in the LSC, total methylation (defined as the cumulative prevalence of methylation for all 11 genes; see Methods) was significantly higher in smokers with CMH, as was methylation prevalence of SULF2, JPH3, and PCDH20 (p < 0.05, all analyses) (Table 3). Similarly, adjusted analysis in the PLuSS showed that total methylation was significantly higher in those with CMH, as was methylation prevalence of *SULF2*, p16, and *PCDH20* (p < 0.05, all analyses) (Table 3).

**Table 3:** Odds ratios for CMH in adjusted* **analyses**

	**LSC n = 900**			**PLuSS n = 490**			**Combined n = 1390**		
**Exposure variable**	**OR**	**(95 % CL)**	**p value**	**OR**	**(95 % CL)**	**p value**	**OR**	**(95 % CL)**	**p value**
**Total methylation**	1.09***	(1.02, 1.17)	**0.014**	1.15	(1.03, 1.29)	**0.014**	1.11	(1.04, 1.18)	0.001**
**SULF2**	1.68	(1.23, 2.30)	0.001**	2.14	(1.38, 3.31)	0.001**	1.79	(1.39, 2.31)	<.0001**
**p16**	1.43	(0.97, 2.11)	0.067	1.69	(1.01, 2.83)	**0.045**	1.54	(1.14, 2.10)	**0.006**
**JPH3**	1.45	(1.04, 2.03)	**0.031**	1.70	(0.83, 3.48)	0.149	1.53	(1.13, 2.08)	**0.006**
**PCDH20**	1.40	(1.03, 1.90)	**0.033**	1.73	(1.10, 2.73)	**0.018**	1.47	(1.14, 1.90)	0.003**

Each row represents a separate adjusted model.

LSC: CMH (n = 311) and no CMH (n = 589); PLuSS CMH (n = 140) and no CMH (n = 350).

*Additional adjustors include age, sex, education, COPD, current smoking, pack years, and cohort (for combined analysis); CMH as outcome.

**Bonferroni adjusted significance threshold is p < 0.0042.

***Represents the odds ratio increase for each additional gene methylated.

Analyses combining the two cohorts were also performed. In both unadjusted (Additional file 1: Table S1) and adjusted (Table 3) analysis in the combined cohorts, total methylation was higher in those with CMH than in those with an absence of CMH, as was methylation prevalence of *SULF2*, *JPH3*, *p16* and *PCDH20* (p < 0.01, all analyses). Additional factors associated with CMH were younger age, less education, having COPD, greater pack years, and current smoking (p < 0.01, all analyses, Additional file 1: Table S1). Additional modeling was performed that included two-way interaction terms for baseline COPD, pack years and methylation, total or individual gene for the combined cohort of LSC and PLuSS cohorts. These interaction terms were not significant for total methylation, Sulf-2, or PCDH20, each of which showed significant association with CMH within the LSC, the PluSS cohort, and the combination of both cohorts. These findings suggest methylation is an independent risk for CMH.

### The association between CMH and gene promoter methylation is stronger in males

Univariate analysis revealed factors that were associated with higher methylation prevalence, which include male sex (p < 0.001) (Additional file 1: Table S2). Because of the observed sex differences in methylation prevalence, sex stratified analyses were performed in males and females. Total methylation was significantly associated with CMH in males in both the LSC and PLuSS cohorts (p < 0.01, both analyses) and when analysis was performed for the combined cohort (p < 0.001) (Table 4). When individual genes were analyzed in males, *SULF2*, p16, and *JPH3* were significantly associated in the LSC (p < 0.05, all analyses), while SULF2 and PCDH20 were significant in the PLuSS (p < 0.05). In the combined cohort, the prevalence of *SULF2*, *JPH3*, *PCDH20*, and *p16* methylation were all significantly higher in males with CMH compared to males without CMH (p < 0.05, all analyses). Although the number of female participants was higher for both cohorts, in females, no significant associations were found for the individual cohort analyses, although higher SULF2 methylation prevalence was observed in analysis of the combined cohorts (p < 0.05).

**Table 4:** Odds ratios for CMH in sex stratified adjusted* **analyses**

**Males**	**LSC n = 227**			**PLuSS n = 183**			**Combined n = 410**		
**Exposure variable**	**OR**	**(95 % CL)**	**p value**	**OR**	**(95 % CL)**	**p value**	**OR**	**(95 % CL)**	**p value**
**Total methylation**	1.23	(1.07, 1.41)	0.004**	1.28	(1.07, 1.54)	**0.008**	1.23	(1.11, 1.37)	<.0001**
**SULF2**	2.72	(1.51, 4.91)	0.001**	2.97	(1.48, 5.95)	0.002**	2.73	(1.75, 4.25)	<.0001**
**p16**	2.08	(1.01, 4.28)	**0.048**	1.66	(0.71, 3.89)	0.246	1.88	(1.09, 3.23)	**0.023**
**JPH3**	2.64	(1.43, 4.87)	0.002**	2.70	(0.96, 7.59)	0.059	2.66	(1.58, 4.48)	<.0001**
**PCDH20**	1.68	(0.94, 2.98)	0.079	2.29	(1.15, 4.53)	**0.018**	1.89	(1.22, 2.93)	0.004**
**Females**	**LSC n = 673**			**PLuSS n = 307**			**Combined n = 980**		
**Exposure variable**	**OR**	**(95 % CL)**	**p value**	**OR**	**(95 % CL)**	**p value**	**OR**	**(95 % CL)**	**p value**
**Total methylation**	1.04	(0.96, 1.14)	0.324	1.08	(0.93, 1.25)	0.322	1.05	(0.98, 1.13)	0.188
**SULF2**	1.41	(0.97, 2.05)	0.074	1.72	(0.96, 3.07)	0.066	1.47	(1.07, 2.01)	**0.016**
**p16**	1.25	(0.79, 1.99)	0.342	1.76	(0.91, 3.40)	0.091	1.41	(0.97, 2.05)	0.073
**JPH3**	1.07	(0.71, 1.62)	0.748	1.20	(0.42, 3.39)	0.736	1.10	(0.75, 1.62)	0.634
**PCDH20**	1.33	(0.92, 1.93)	0.126	1.35	(0.72, 2.52)	0.346	1.34	(0.97, 1.83)	0.073

Each row represents a separate adjusted model.

*Additional adjustors include age, education, COPD, current smoking, pack years, and cohort (for combined analysis); CMH as outcome.

**Bonferroni adjusted significance threshold is p < 0.0042.

### The association between CMH and gene promoter methylation is stronger in former smokers

Current smoking status and pack years were controlled for in adjusted analyses (Tables 3 and 4); however, residual confounding remains a possibility, given that current smoking strongly influences CMH status (Tables 1 and 2). Therefore, stratified analyses were performed in current and former smokers. Adjusted stratified analysis revealed for both the LSC and PLuSS that total methylation was significantly higher in those with CMH who were former smokers (p < 0.05, all analyses) (Table 5). Although the number of current smokers was greater in both cohorts, in current smokers total methylation was not significantly associated with CMH in either cohort or the combined analysis. In general, the associations between methylation and CMH were less significant and demonstrated smaller effect sizes in current smokers (Table 5). Sex and smoking stratified analysis of the combined cohorts (combined to ensure adequate sample size for analysis) (Table 6) revealed that the strongest associations between methylation and CMH were observed in male former smokers, with odds ratios for the individual genes ranging from 2.55 to 4.34. Despite 2-3-fold greater number of female participants in the LSC and PLuSS, only SULF-2 methylation was associated with CMH in females from the combined cohorts.

**Table 5:** Odds ratios for CMH in adjusted* **analyses of current and former smokers**

	**LSC former smokers n = 406**			**PLuSS former smokers n = 155**			**Combined former smokers n = 561**		
**Exposure variable**	**OR**	**(95 % CL)**	**p value**	**OR**	**(95 % CL)**	**p value**	**OR**	**(95 % CL)**	**p value**
**Total methylation**	1.13	(1.01, 1.26)	**0.034**	1.36	(1.10, 1.67)	0.004**	1.18	(1.07, 1.30)	0.001**
**SULF2**	1.96	(1.17, 3.29)	**0.011**	3.64	(1.51, 8.77)	0.004**	2.30	(1.47, 3.59)	<.0001**
**p16**	2.06	(1.13, 3.76)	**0.018**	1.53	(0.59, 3.95)	0.377	1.92	(1.16, 3.17)	**0.012**
**JPH3**	1.79	(1.04, 3.09)	**0.037**	2.76	(0.99, 7.71)	0.053	2.02	(1.25, 3.26)	0.004**
**PCDH20**	1.9	(1.13, 3.18)	**0.015**	1.15	(0.46, 2.90)	0.768	1.67	(1.07, 2.61)	**0.024**
	**LSC current smokers n = 494**			**PLuSS current smokers n = 335**			**Combined current smokers n = 829**		
**Exposure variable**	**OR**	**(95 % CL)**	**p value**	**OR**	(**95 % CL)**	**p value**	**OR**	**(95 % CL)**	**p value**
**Total methylation**	1.07	(0.97, 1.17)	0.18	1.07	(0.94, 1.23)	0.303	1.07	(0.99, 1.15)	0.106
**SULF2**	1.53	(1.03, 2.28)	**0.036**	1.82	(1.09, 3.04)	**0.022**	1.60	(1.17, 2.19)	0.003**
**p16**	1.15	(0.70, 1.89)	0.578	1.83	(0.98, 3.41)	0.057	1.39	(0.95, 2.05)	0.094
**JPH3**	1.3	(0.84, 1.99)	0.237	1.16	(0.42, 3.19)	0.779	1.29	(0.87, 1.91)	0.200
**PCDH20**	1.17	(0.79, 1.72)	0.433	1.98	(1.16, 3.36)	**0.012**	1.39	(1.02, 1.90)	**0.039**

Each row represents a separate adjusted model.

*Additional adjustors include age, sex, education, COPD, pack years, and cohort (for combined analysis); CMH as outcome.

**Bonferroni adjusted significance threshold is p < 0.0042.

**Table 6:** Odds ratios for CMH in sex stratified adjusted* **analyses of current and former smokers**

**Males**	**Current smokers n** = **269**			**Former smokers n** = **139**		
**Exposure variable**	**OR**	**(95 % CL)**	**p value**	**OR**	**(95 % CL**)	**p value**
**Total methylation**	1.13	(0.99, 1.30)	0.076	1.39	(1.16, 1.67)	<.0001**
**SULF2**	2.52	(1.47, 4.32)	0.001**	3.65	(1.59, 8.37)	0.002**
**p16**	1.36	(0.69, 2.67)	0.378	3.69	(1.42, 9.60)	**0.007**
**JPH3**	1.97	(1.02, 3.79)	**0.043**	4.34	(1.74, 10.79)	0.002**
**PCDH20**	1.71	(1.02, 2.86)	**0.043**	2.55	(1.11, 5.83)	**0.027**
**Females**	**Current smokers n** = **560**			**Former smokers n** = **410**		
**Exposure variable**	**OR**	**(95 % CL)**	**p value**	**OR**	**(95 % CL)**	**p value**
**Total methylation**	1.02	(0.93, 1.12)	0.617	1.10	(0.97, 1.24)	0.142
**SULF2**	1.27	(0.86, 1.88)	0.221	1.92	(1.12, 3.29)	**0.019**
**p16**	1.34	(0.83, 2.16)	0.230	1.52	(0.83, 2.80)	0.178
**JPH3**	0.94	(0.57, 1.56)	0.817	1.40	(0.77, 2.55)	0.272
**PCDH20**	1.28	(0.87, 1.89)	0.217	1.40	(0.81, 2.42)	0.226

Each row represents a separate adjusted model.

Analysis performed on combined cohorts.

*Additional adjustors include age, education, COPD, pack years, and cohort; CMH as outcome.

**Bonferroni adjusted significance threshold is p < 0.0042.

### Sputum methylation is a sensitive and specific predictor of CMH in male former smokers

**Figure 1:**
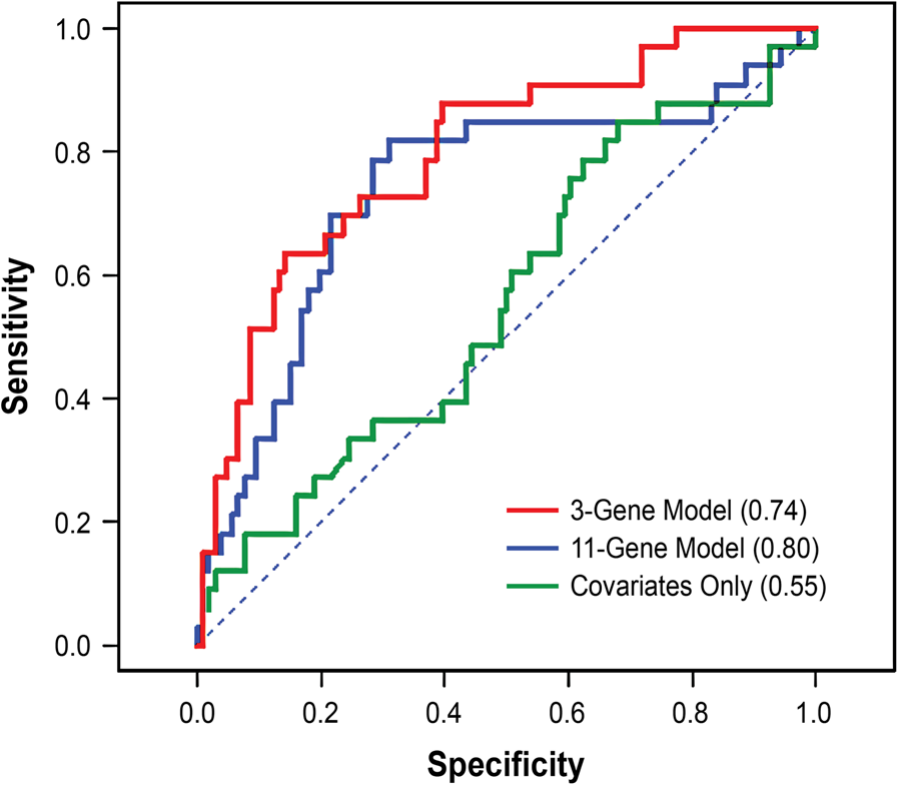
**ROC curves comparing the sensitivity and specificity of 3**- **and 11**-**gene methylation panels for classifying CMH.** ROC curves were generated by applying logistic regression models to male former smokers (n = 139) from the combined PLuSS and LSC. The covariates included age, pack years, education, and COPD. AUC is indicated in parentheses.

Receiver operator characteristic (ROC) curves were generated to assess the sensitivity and specificity of logistic regression models for discriminating CMH. Prior to generating ROC curves, modeling was performed to assess all combinations of predictors, including all 11 genes and covariates. The Akaike information content (AICc) [25] was used to select the models with an optimal trade-off between accuracy and complexity. Independently in both the LSC and the PLuSS, the best-fitting model was a 3-gene model that included *SULF2*, *JPH3*, and *p16* as predictors, as well as age, pack years, education, and COPD (data not shown). Therefore, using the combined sample from the LSC and PLuSS, ROC curves were generated using the 3-gene model, the full 11-gene model, and covariates-only model in male former smokers (Figure 1). Likelihood ratio tests confirmed that both the 3-gene and 11-gene models are significantly more discriminative than the covariates only model (p = 0.0002 and p = 0.002, respectively); however, the 3- and 11-gene models were not significantly different from each other (p = 0.29). Areas under the curve (AUC) were 0.74 and 0.80 for the 3- and 11-gene models, respectively, while the AUC was 0.55 for the covariates only model. Although sample sizes were small in cohort-stratified analyses of male former smokers, these analyses demonstrate that the increased discriminative power of the 3-gene model is observed in two independent cohorts (Additional file 1: Figure S1).

## Discussion

This study demonstrates a significant association between CMH and prevalence of promoter methylation in sputum of lung cancer risk genes in two geographically distinct cohorts. This association was especially strong in males and in former smokers, and SULF2 was the most consistently associated gene. Importantly, the overall association between CMH and methylation, and the specific effects of sex and smoking status, were observed independently in both cohorts. Combining the two cohorts strengthened the statistical significance of these associations. The central finding of our study is that male former smokers with unresolved CMH may be at an increased risk of lung cancer. Given that 50% of persons diagnosed with lung cancer are former smokers, prospective studies evaluating the methylation status of former smokers with CMH who subsequently develop lung cancer are needed [26].

The eleven genes examined in this study were selected based on prior evidence that they are associated with lung cancer risk [17,18]. Therefore, increased prevalence of methylation of these genes may predict lung cancer among subjects with CMH. These gene promoters have all been shown to be methylated in tumors [27,28], and are proposed to represent an expanding field of precancerous epigenetic changes in the aerodigestive tract of smokers [17,29]. This hypothesis is supported by the observation that the methylation prevalence of these gene promoters increases as the time to lung cancer diagnosis decreases [17]. Mounting evidence indicates that these changes are causal for tumor initiation [30-33].

The association between methylation and CMH was markedly stronger in males than in females (Table 4). Univariate analysis of males and females in both cohorts (Additional file 1: Tables S3 and S4) reveals that females with CMH are significantly younger than female controls in the LSC; however this was not true in the PLuSS. Additionally age was a covariate in all adjusted analyses and thus is unlikely to account for the lack of association between methylation and CMH in females. This apparent protective mechanism in females warrants further study. The association between methylation and CMH was also stronger in former than in current smokers (Table 5). The increase in effect size in former smokers may be due to several reasons: (1) the CMH phenotype in former smokers may not be confounded by cough and phlegm caused by irritation due to current smoking; (2) in susceptible smokers, CMH that persists in spite of smoking cessation may represent a phenotype with a more distinct molecular pathology; (3) The association between CMH and gene promoter methylation may be stronger with age. In the LSC and PLuSS cohorts, former smokers were significantly older than current smokers (mean age difference 4.2 years, data not shown). This age difference between former and current smokers also likely explains the puzzling observation that current smokers have lower overall methylation compared to former smokers (Additional file 1: Table S2); current smokers are younger, and younger age is associated with less total methylation in these lung cancer risk genes.

Numerous studies have demonstrated that prior CMH significantly increases the risk for later development of lung cancer (reviewed in [15,16]). Assessment of the latency period between diagnosis of CMH and diagnosis of lung cancer has shown that this risk increases with time since diagnosis of CMH [34]. In one study [34], the odds ratio nearly quadrupled at latency >15 years compared to latency 1–5 years. Importantly, this suggests that CMH may serve as a precursor to lung carcinogenesis [34]. We hypothesize that the increased prevalence for methylation of the lung cancer risk genes seen in this study may help explain the epidemiological link between CMH and lung cancer. Further studies are needed to establish a direct link between gene methylation and lung cancer. Interestingly, while SULF2, p16, JPH3, and PCDH20 all demonstrate evidence for association with CMH in the current study, a previous study determined that GATA4 promoter methylation was associated with airflow obstruction [35]. These findings suggest that major differences exist in the genes affected by aberrant promoter methylation in distinct COPD sub-phenotypes. This is consistent with the major pathophysiological differences that underlie emphysema and chronic mucous hypersecretion [36], and suggests the role basal cell hyperplasia may play in development of lung cancer [37].

Of the 11 genes analyzed, SULF2 demonstrated the strongest association with CMH. SULF-2 is an extracellular enzyme that catalyzes the hydrolysis of 6-O-sulfo groups from heparan sulfate polysaccharides [38-40]. Heparan sulfate proteoglycans (HSPGs) are widely distributed on cell membranes and the extracellular matrix, and serve as coreceptors for many growth factors and cytokines [41] and the position of 6-O sulfates is of particular importance for ligand binding [38-40]. Epigenetic inactivation of SULF2, either by siRNA treatment or promoter methylation, activates numerous type I interferon (IFN)-inducible genes [42]. It was proposed that silencing of SULF2 prevents the removal of sulfate groups from IFN-binding sites, which may preserve either the binding affinity or bioavailability of interferons leading to increased transcription of multiple IFN-inducible genes [42]. It is plausible that CMH, caused by metaplastic mucous cells that are sustained due to dysregulated cell death mechanisms that involve IFN signaling [43-45], creates an inflammatory milieu which causes methylation of SULF2. In turn, the type 1 interferon response induced by methylation of SULF2 may help to perpetuate the inflammation associated with CMH.

This is the first report of epigenetic changes in the airways of individuals with CMH. Strengths of the study include the use of the large, well-characterized LSC for the initial phase of study and excellent replication of all main findings in the geographically distinct PLuSS. We chose the standard definition for chronic bronchitis in the LSC and a definition that most closely captured the standard clinical definition of chronic bronchitis in the PLuSS. While the differences in questionnaires used to define CMH could be considered a limitation in the study, the definition for CMH was applied to PLuSS subjects prior to any data analysis and was not subsequently modified. We propose that this approach improves the rigor of our validation. Replication of these findings supports the robustness of these markers for CMH and suggests that they are useful in defining a subset of subjects with CMH who could benefit from computed tomography (CT) screening for lung cancer [46]. Indeed, low cost, gene-specific methylation screening assays could be incorporated into clinical practices for patients suspected to be at risk for lung cancer.

## Conclusions

Especially male former smokers with persistent chronic mucous hypersecretion have markedly increased promoter methylation of lung cancer risk genes in cell obtained by sputum collection. These smokers may be at increased risk of lung cancer and may benefit from further tests for lung cancer, such as CT screening.

## Competing interests

The authors declare that they have no competing interests.

## Authors’ contributions

YT, SB, and HP made substantial contributions to conception and design; SB, JW, JS, HP, SB, and MP made substantial contributions to acquisition of data or analysis and interpretation of data. All authors made substantial contributions to drafting the article or revising it critically for important intellectual content and final approval of the version to be published.

## Supplementary Material

Additional file 1: Table S1 Select variables by CMH status in the combined cohorts. **Table S2.** Select variables by high and low methylation tertile in combined cohorts. **Table S3.** Select variables by CMH in males from the LSC and PLuSS. **Table S4.** Select variables by CMH in females from the LSC and PLuSS. **Figure S1.** ROC curves comparing the sensitivity and specificity of the 3-gene methylation panels for classifying CMH. ROC curves were generated by applying logistic regression models to male former smokers independently in the PLuSS (n = 52) and LSC (n = 87). The covariates included age, pack years, education and COPD. AUC is indicated in parentheses. Click here for file
