# Supplementary material for: Increased methylation of lung cancer-associated genes in sputum DNA of former smokers with chronic mucous hypersecretion
Source: Respir Res. 2014 Jan 9;15(1):2. doi: 10.1186/1465-9921-15-2 (PMC3893562; doi:10.1186/1465-9921-15-2)
Supplement: Additional file 1: Table S1 — Select variables by CMH status in the combined cohorts. Table S2. Select variables by high and low methylation tertile in combined cohorts. Table S3. Select variables by CMH in males from the LSC and PLuSS. Table S4. Select variables by CMH in females from the LSC and PLuSS. Figure S1. ROC curves comparing the sensitivity and specificity of the 3-gene methylation panels for classifying CMH. ROC curves were generated by applying logistic regression models to male former smokers independently in the PLuSS (n = 52) and LSC (n = 87). The covariates included age, pack years, education and COPD. AUC is indicated in parentheses. [file 1465-9921-15-2-S1.docx]

**Supplementary Information**

**Increased Methylation of Lung Cancer-Associated Genes in Sputum DNA of Former Smokers with Chronic Mucous Hypersecretion**

Shannon Bruse^1a^, Hans Petersen^1a^, Joel Weissfeld^2^, Maria Picchi^1^, Randall Willink^1^, Kieu Do^1^, Jill Siegfried^2^, Steven A. Belinsky^1^, Yohannes Tesfaigzi^1^

Institutional Affiliations:

^1^ Lovelace Respiratory Research Institute, Albuquerque, NM;

^2^Department of Pharmacology & Chemical Biology, Hillman Cancer Center of the University of Pittsburgh Medical Center, Pittsburgh, PA,

*Authors contributed equally to this work

| **Supplementary Table 1: Select variables by CMH status in the combined cohorts** | | | | | | | |
| --- | --- | --- | --- | --- | --- | --- | --- |
| **Combined** | **Total** | | **CMH1** | | **No CMH** | |  |
|  | **n=1390** | **(100.0)** | **n=451** | **(100.0)** | **n=939** | **(100.0)** |  |
| **Characteristic** | **N or mean** | **(%) or (SD)** | **N or mean** | **(%) or (SD)** | **N or mean** | **(%) or (SD)** | **p value** |
| **Female** | 980 | (70.5) | 312 | (69.2) | 668 | (71.1) | 0.453 |
| **Baseline Age** | 57.4 | (8.8) | 56.5 | (9.1) | 57.9 | (8.7) | **0.004** |
| **Education >= HS** | 1137 | (81.8) | 341 | (75.6) | 796 | (84.8) | **<.0001** |
| **Obese** | 436 | (31.4) | 138 | (30.6) | 298 | (31.7) | 0.669 |
| **Pack Years** | 46.3 | (21.8) | 48.8 | (22.1) | 45.1 | (21.6) | **0.003** |
| **Baseline Smoker** | 829 | (60.2) | 349 | (77.7) | 480 | (51.7) | **<.0001** |
| **Baseline COPD** | 519 | (37.3) | 204 | (45.2) | 315 | (33.6) | **<.0001** |
| **Total Methylation** | 2.31 | (2.04) | 2.53 | (2.17) | 2.2 | (1.96) | **0.004** |
| **PCDH20** | 468 | (33.7) | 172 | (38.1) | 296 | (31.5) | **0.015** |
| **SULF2** | 446 | (32.1) | 178 | (39.5) | 268 | (28.5) | **<.0001** |
| **GATA4** | 514 | (37.0) | 172 | (38.1) | 342 | (36.4) | 0.535 |
| **PAX5A** | 206 | (14.8) | 69 | (15.3) | 137 | (14.6) | 0.728 |
| **p16** | 246 | (17.7) | 93 | (20.6) | 153 | (16.3) | **0.048** |
| **MGMT** | 372 | (26.8) | 121 | (26.8) | 251 | (26.7) | 0.969 |
| **DAPK** | 228 | (16.4) | 75 | (16.6) | 153 | (16.3) | 0.874 |
| **GATA5** | 216 | (15.5) | 73 | (16.2) | 143 | (15.2) | 0.645 |
| **PAX5B** | 123 | (8.9) | 39 | (8.7) | 84 | (9.0) | 0.855 |
| **DAL1** | 108 | (7.8) | 41 | (9.1) | 67 | (7.1) | 0.202 |
| **JPH3** | 270 | (19.4) | 106 | (23.5) | 164 | (17.5) | **0.008** |

| **Supplementary Table 2: Select variables by high and low methylation tertile in combined cohorts** | | | | | | | |
| --- | --- | --- | --- | --- | --- | --- | --- |
|  | **Total Methylation /HiT/Lo** | | **Total Methylation - HiTert** | | **Total Methylation - LoTert** | |  |
|  | **n=1112** | **(100.0)** | **n=531** | **(100.0)** | **n=581** | **(100.0)** |  |
| **Characteristic** | **N or mean** | **(%) or (SD)** | **N or mean** | **(%) or (SD)** | **N or mean** | **(%) or (SD)** | **p value** |
| **Female** | 796 | (71.58) | 341 | (64.22) | 455 | (78.31) | **<.0001** |
| **Male** | 316 | (28.42) | 190 | (35.78) | 126 | (21.69) | **<.0001** |
| **Baseline Age** | 57.4 | (9.00) | 58.3 | (9.14) | 56.7 | (8.80) | **0.003** |
| **Education >= HS** | 900 | (80.94) | 419 | (78.91) | 481 | (82.79) | 0.100 |
| **Obese** | 341 | (30.67) | 172 | (32.39) | 169 | (29.09) | 0.233 |
| **Pack Years** | 46.2 | (21.81) | 46.9 | (22.34) | 45.6 | (21.32) | 0.330 |
| **Baseline Smoker** | 658 | (59.76) | 287 | (54.25) | 371 | (64.86) | **0.000** |
| **Baseline CMH1** | 375 | (33.72) | 198 | (37.29) | 177 | (30.46) | **0.016** |

**Supplementary Table 3: Select variables by CMH in males from the LSC and PLuSS**

|  | **LSC Males** | | | | | **PLuSS Males** | | | | |
| --- | --- | --- | --- | --- | --- | --- | --- | --- | --- | --- |
|  | **CMH** | | **No CMH** | |  | **CMH** | | **No CMH** | |  |
| **Characteristic** | **89** | **(100.0)** | **138** | **(100.0)** |  | **50** | **(100.0)** | **133** | **(100.0)** |  |
|  | **N or mean** | **(%) or (SD)** | **N or mean** | **(%) or (SD)** | **p value** | **N or mean** | **(%) or (SD)** | **N or mean** | **(%) or (SD)** | **p value** |
| **Baseline Age** | 56.10 | (9.6) | 56.40 | (9.7) | 0.822 | 60.04 | (5.1) | 60.74 | (5.3) | 0.423 |
| **Education >= HS** | 66 | (74.2) | 110 | (79.7) | 0.328 | 48 | (96.0) | 127 | (95.5) | 0.880 |
| **Obese** | 25 | (28.1) | 42 | (30.4) | 0.705 | 20 | (40.0) | 49 | (36.8) | 0.695 |
| **Pack Years** | 48.66 | (21.9) | 43.02 | (20.6) | 0.051 | 67.94 | (18.0) | 62.29 | (18.4) | 0.064 |
| **Baseline Smoker** | 67 | (75.3) | 73 | (52.9) | **0.001** | 39 | (78.0) | 92 | (69.2) | 0.238 |
| **Baseline COPD** | 48 | (53.9) | 47 | (34.1) | **0.003** | 32 | (64.0) | 68 | (51.1) | 0.119 |
| **FEV1/FVC** | 67.22 | (11.7) | 71.99 | (8.8) | **0.001** | 62.71 | (13.0) | 66.45 | (10.9) | 0.052 |
| **Total Methylation** | 3.61 | (2.3) | 2.75 | (2.1) | **0.004** | 2.96 | (2.2) | 2.11 | (1.7) | **0.007** |
| **Total St Geo** | 28.14 | (18.0) | 16.60 | (14.6) | **<.0001** | 27.12 | (15.6) | 15.64 | (14.7) | **<.0001** |
| **Symptom subscale** | 48.08 | (20.5) | 27.33 | (21.3) | **<.0001** | 48.26 | (18.8) | 24.70 | (18.2) | **<.0001** |
| **Impacts subscale** | 15.69 | (14.4) | 8.15 | (10.6) | **<.0001** | 17.34 | (14.7) | 8.76 | (12.2) | **<.0001** |
| **Activity subscale** | 34.42 | (25.5) | 23.35 | (21.4) | **0.001** | 31.96 | (22.0) | 21.75 | (21.2) | **0.005** |

**Supplementary Table 4: Select variables by CMH in females from the LSC and PLuSS**

|  | **LSC Females** | | | | | **PLuSS Females** | | | | |
| --- | --- | --- | --- | --- | --- | --- | --- | --- | --- | --- |
|  | **CMH** | | **No CMH** | |  | **CMH** | | **No CMH** | |  |
| **Characteristic** | **222** | **(100.0)** | **451** | **(100.0)** |  | **90** | **(100.0)** | **217** | **(100.0)** |  |
|  | **N or mean** | **(%) or (SD)** | **N or mean** | **(%) or (SD)** | **p value** | **N or mean** | **(%) or (SD)** | **N or mean** | **(%) or (SD)** | **p value** |
| **Baseline Age** | 54.67 | (9.9) | 56.27 | (9.3) | **0.039** | 59.21 | (6.6) | 60.59 | (7.1) | 0.117 |
| **Education >= HS** | 141 | (63.5) | 346 | (76.7) | **0.000** | 86 | (95.6) | 213 | (98.2) | 0.193 |
| **Obese** | 68 | (30.6) | 143 | (31.7) | 0.777 | 25 | (27.8) | 64 | (29.5) | 0.763 |
| **Pack Years** | 42.41 | (21.5) | 38.39 | (20.0) | **0.017** | 54.03 | (18.0) | 49.91 | (20.2) | 0.095 |
| **Baseline Smoker** | 169 | (76.1) | 192 | (42.6) | **<.0001** | 74 | (82.2) | 130 | (59.9) | **0.000** |
| **Baseline COPD** | 75 | (33.8) | 111 | (24.6) | **0.012** | 49 | (54.4) | 89 | (41.0) | **0.031** |
| **FEV1/FVC** | 71.36 | (11.4) | 73.76 | (10.3) | **0.006** | 66.83 | (12.2) | 69.69 | (11.4) | 0.051 |
| **Total Methylation** | 2.27 | (2.1) | 2.26 | (2.0) | 0.927 | 1.87 | (1.9) | 1.77 | (1.7) | 0.678 |
| **Total St Geo** | 32.85 | (19.2) | 17.59 | (15.2) | **<.0001** | 33.18 | (18.4) | 15.65 | (13.9) | **<.0001** |
| **Symptom subscale** | 49.01 | (21.2) | 23.04 | (19.7) | **<.0001** | 53.45 | (18.1) | 24.16 | (16.9) | **<.0001** |
| **Impacts subscale** | 19.92 | (17.4) | 8.45 | (11.2) | **<.0001** | 21.89 | (18.0) | 7.68 | (11.4) | **<.0001** |
| **Activity subscale** | 42.18 | (25.0) | 28.56 | (23.3) | **<.0001** | 40.74 | (25.9) | 23.83 | (22.2) | **<.0001** |

**Supplementary Figure 1:** ROC curves comparing the sensitivity and specificity of the 3-gene methylation panels for classifying CMH. ROC curves were generated by applying logistic regression models to male former smokers independently in the PLuSS (n=52) and LSC (n=87). The covariates included age, pack years, education and COPD. AUC is indicated in parentheses.
